# SA-FEM: Combined Feature Selection and Feature Fusion for Students’ Performance Prediction

**DOI:** 10.3390/s22228838

**Published:** 2022-11-15

**Authors:** Mingtao Ye, Xin Sheng, Yanjie Lu, Guodao Zhang, Huiling Chen, Bo Jiang, Senhao Zou, Liting Dai

**Affiliations:** 1 Department of Digital Media Technology, Hangzhou Dianzi University, Hangzhou 310018, China; 2 Huannan Subdistrict Office, Dinghai District, Zhoushan 316000, China; 3 College of Computer Science and Artificial Intelligence, Wenzhou University, Wenzhou 325000, China; 4 Shanghai Institute of AI Education, East China Normal University, Shanghai 200062, China; 5 College of Computer Science and Technology (College of Artificial Intelligence), Zhejiang Sci-Tech University, Hangzhou 310018, China

**Keywords:** e-learning behavior classification, feature selection, feature fusion, prediction of learning performance, prediction and analysis

## Abstract

Around the world, the COVID-19 pandemic has created significant obstacles for education, driving people to discover workarounds to maintain education. Because of the excellent benefit of cheap-cost information distribution brought about by the advent of the Internet, some offline instructional activity started to go online in an effort to stop the spread of the disease. How to guarantee the quality of teaching and promote the steady progress of education has become more and more important. Currently, one of the ways to guarantee the quality of online learning is to use independent online learning behavior data to build learning performance predictors, which can provide real-time monitoring and feedback during the learning process. This method, however, ignores the internal correlation between e-learning behaviors. In contrast, the e-learning behavior classification model (EBC model) can reflect the internal correlation between learning behaviors. Therefore, this study proposes an online learning performance prediction model, SA-FEM, based on adaptive feature fusion and feature selection. The proposed method utilizes the relationship among features and fuses features according to the category that achieved better performance. Through the analysis of experimental results, the feature space mined by the fine-grained differential evolution algorithm and the adaptive fusion of features combined with the differential evolution algorithm can better support online learning performance prediction, and it is also verified that the adaptive feature fusion strategy based on the EBC model proposed in this paper outperforms the benchmark method.

## 1. Introduction

As COVID-19 spread rapidly around the world since 2019, the normal operations of educational institutions were more seriously affected, and offline teaching was shifted to online in order to prevent the spread of the pandemic and ensure the progress of teaching [1]. At the same time, the development of the Internet and its advantage of disseminating information is the inevitable trend of online education. Due to the unevenness of regional development, educational resources also tend to gather in regions with good economic development areas [2], and online learning also helps to alleviate the general environment of educational inequity. Online education in China is late compared to developed countries, so it has more room for development. In the process of conducting online teaching, teachers and students may be inexperienced in online education and are unable to provide real-time feedback to each other, leading to uneven teaching quality and eventually to student dropout [3]. How to guarantee the quality of e-learning and reduce the overall dropout rate is an urgent issue. 

The development of online learning needs the help of platform equipment. When many learners use various online learning platforms for online learning, a large amount of learning behavior data are collected and recorded. Many researchers have done much work on improving the quality of learners’ online learning. For example, Marras et al. extracted a new feature set based on online learning behavior data and revealed that the performance of student performance prediction is highly dependent on learning behavior [4]. Using these behavioral data, such as video clickstream data and assignment submission behavior data, can maximize the value of learning behavior data and improve the prediction of online learning performance. He et al. [5] proposed a joint RNN-GRU neural network approach to fit static and sequential data in the Open University Learning Analytics Dataset (OULAD), which showed high accuracy in predicting at-risk students. Hao et al. [6] constructed a Bayesian network, SPBN, for student performance prediction by the hill-climbing method and maximum likelihood estimation. The experimental results on OULAD show the accuracy of the SPBN in predicting student performance. Such methods show better performance based on independent learning behavior data. The predicted results help teachers correct teaching methods, promptly provide feedback on teaching effectiveness, inform students to adjust their learning status, and serve as a monitoring and feedback function.

The aforementioned methods can produce accurate predictions, but they have certain drawbacks. First, the use of manual methods for data pre-processing has the problem of low efficiency when facing a large amount of learner behavior data. Second, the operation is generally crude when using online learning prediction models for feature space construction. It usually classifies all the learning features directly based on the algorithm and then uses them for learning performance prediction. Third, the learning behavior features tend to be homogeneous and ignore the intrinsic connection of learning behaviors. Online learning behaviors do not occur independently, regardless of the order in which they occur or the correlation between learning behaviors. Reducing the time and labor cost of data pre-processing and considering the correlation between behaviors for feature dimensionality reduction is a key problem that needs to be urgently solved.

In conclusion, this paper proposes an online learning performance prediction model, SA-FEM, with adaptive feature fusion and selection to further optimize the effectiveness of online learning performance prediction. The main contributions are as follows:

- This paper proposes an end-to-end learning performance prediction model. First, we construct a wrapper method using differential evolution algorithms as an evaluation function to implement feature selection. Second, we further use the e-learning behavior classification model to fuse the preliminary selected features with finer granularity. While reducing the computational load of the model, the feature fusion based on the learning process behavior classification model takes into account the correlation between the behavior features and fuses the same category of features as the input of the prediction model, which greatly enhances the interpretability of the model;
- This paper presents an adaptive feature fusion method. Firstly, through data visualization, the distribution rules of different types of learners are observed. Secondly, according to the discreteness of each learner’s distribution, different feature fusion strategies are formulated. Finally, the cluster similarity is used to identify the learner categories, and the corresponding feature fusion strategies are selected adaptively;
- In the aspect of advanced validation of mechanism construction, through a large number of experimental comparisons, it is found that the ablation experiments of feature fusion show that the performance and efficiency of the model can be effectively improved after feature fusion. Comparative experiments of the different mutation strategies show the advancement of our differential evolution algorithm.
- In terms of validation of algorithm advancement, an experimental analysis on the course FFF of the Open University Learning Analytics Dataset (OULAD) demonstrates the validity of the SA-FEM proposed in this paper, and it is superior to the benchmark method in model performance, such as accuracy, F1 score, etc.

The rest of the article is organized as follows: Section 2 introduces predictors of e-learning behavior and methods for predicting learner performance; Section 3 describes our proposed model for predicting learning performance; Section 4 presents the experimental design and evaluation metrics; Section 5 discusses the experimental results; and Section 6 gives conclusions and an introduction to future work.

## 2. Related Work

We reviewed research on e-learning behavior and categorized e-learning performance features into two categories: dispositional features and behavioral features. Dispositional features are inherent properties of learners themselves, which are generally static data and mostly private and cannot be shared publicly; behavioral features are dynamic features of learners in the learning process, such as collaborative communication and forum discussions, and can be a good indicator of the time and effort students spend on a particular course.

Because behavioral performance features do not involve privacy, a large number of researchers have invested in this research, and with the improvement of computer computing power and more and more intelligent algorithms, it is possible to predict learners’ online learning performance.

### 2.1. E-Learning Performance Features

Predicting students’ online learning performance has been a hot topic of interest in the field of learning analytics in recent years. Students generate a large amount of data during the process of registering and logging into the platform and performing learning. When using the data recorded and stored in the online learning platform for learning performance prediction, feature engineering operations are often required to make the original data better serve the prediction of online learning performance after pre-processing and feature extraction operations. For example, Wang [7] collected and filtered student behavior data through data preparation, statistics, and analysis, used the apriori algorithm to mine association rules, and calculated data similarity. Finally, they used a fuzzy neural network to mine student behavior data for online English learning. The method achieved high data processing efficiency and low prediction error. Mai et al. [8] proposed a new data pre-processing method for learning behavior data in programming education, which dealt with the noise and trend effect in the data to improve the analysis and prediction based on the learning behavior dataset.

The predictive characteristics of students’ e-learning performance can be grouped into two categories, namely, dispositional and behavioral performance features [9]. Dispositional features are inherent to the learners themselves, such as students’ demographic information [10] and historical education data, mostly static data that are not updated or frequently changed. Many researchers have chosen propensity features to predict students’ learning at various stages of a course or a semester, such as predicting the probability of dropping out of a computer science major by the academic performance in previous courses [11]. However, there are some problems with using propensity features for prediction. First, most propensity features are out of the control of students and instructors, making it difficult to make subsequent changes. Then, there are privacy issues. Propensity traits reflect personally identifiable information and are often difficult to collect and share due to privacy concerns. Finally, the accuracy rate is not high, and it is difficult to achieve good prediction results only through student demographic information and historical education data. For example, machine-learning-based systems identify and select features from personal information, students’ scores, behavioral information, and data from online questionnaires to assess students’ academic performance [12].

Prediction with behavioral performance features generally does not have such a problem. Behavioral performance features, i.e., the dynamic features of learners during the learning process, include online behaviors, textual data, and other multimodal data. These data are easy to collect and easy to change. In online learning, students’ behavioral data are constantly accumulated and updated, and it is important to make online learning performance predictions based on learners’ learning behavior data. Many studies have been conducted to show that students’ online learning behaviors are related to academic performance. For example, Yu et al. [13] used learners’ video clickstream records in a Massive Open Online Course (MOOC) platform to predict learning performance and found a correlation between video viewing behavior and learning outcomes.

However, many researchers have performed learning performance predictions based only on independent online learning behavior data. Loginova et al. [14] embedded learning behavior data and then used RNN to predict student performance. A few researchers also performed feature extraction and selection to achieve more accurate learning performance prediction. Yoo et al. [15] extracted the teaching video viewing behavior of 237 students from LMS and extracted 19 and 21 factors from 157 important predictors using Enet and Mnet to predict the final performance of students. These are predictions based on independent online learning behaviors, and the predictions could be improved. A few researchers also performed feature extraction and selection due to the many sources and types of learning behavior data in online learning to achieve more accurate learning performance prediction. Zheng et al. [16] extracted continuous behavioral features from learners’ learning activity logs. They used the proposed convolutional neural network model FWTS-CNN that fuses feature weighting and behavioral time series to integrate the effects of behavioral features and behavioral time on dropout to improve the accuracy of dropout prediction. Wen et al. [17] proposed a new simple feature matrix to preserve information related to the local relevance of learning behavior and a new convolutional neural network (CNN) model to predict dropouts.

### 2.2. E-Learning Performance Prediction Methods

Online learning platforms store many learning behavior data, such as students’ assignment submission information and video viewing records. More research has proven that having more data does not automatically equate to having better forecasting ability. Akram et al. [18] employed 10 prediction algorithms to identify learning disabilities in pupils by observing how they submitted their assignments. They discovered that as the amount of input data increased, the prediction performance of all algorithms diminished. Therefore, there is a need for more effective methods for learning performance prediction based on a reasonable selection of numerous dynamic features.

The online learning process can be divided into three phases: pre-course, during-course, and end-course. Early pre-course prediction is usually intended to provide hints and warnings. Khan et al. [19] proposed a scalable algorithm called a random wheel for predicting binary and providing desired confidence in prediction to achieve the purpose of predicting failure, degradation, and improvement before the start of the course, allowing the instructor to provide timely help. The algorithm achieved excellent results in numerous datasets. In-class prediction often aims to allow teachers and students to correct promptly, and Syed et al. [20] proposed a gradient-enhanced automatic optimization model to identify and predict students’ procrastination behavior. The model allows teachers to monitor and correct student behavior instantly. Hooshyar et al. [21] used clustering methods to label students as procrastinators, procrastination candidates, or non-procrastinators. Several classification methods were used to classify students and predict student performance by their assignment submission behavior.

Predicting student performance based on learning behavior data as online learning ends can help teachers intervene earlier. Figueroa-Cañas et al. [22] proposed a decision-tree-based model that relies only on average scores on non-mandatory formative assessments to identify students at risk in terms of dropout and final exam performance. Esteban et al. [23] found that using only MIL representation of assignment-related information was more accurate in predicting student academic performance relative to a single-instance learning-based representation. Aydoğdu [24] used artificial neural networks for final student grade prediction through student behavior in navigating and tracking courses in an online learning environment. Mubarak et al. [25] used a long short-term memory network (LSTM) to enable course instructors and educational experts to analyze video clickstream data to allow teachers to develop timely interventions. Song et al. [26] proposed a sequential-engagement-based academic performance prediction network consisting of an engagement detector and a sequential predictor to predict students’ academic performance from their daily learning activity data, historical performance, and demographic information, which has better performance than existing methods when it comes to joining detection mechanism. Hasan et al. [27] used eight different algorithms for data analysis. They used the random forest to accurately predict student performance at the end of the course by reducing features through data transformation techniques, data pre-processing techniques, genetic search, and principal component analysis. Bujang et al. [28] addressed the problem of unbalanced datasets. They proposed an integrated analysis machine learning technique, i.e., a model constructed based on the Synthetic Minority Oversampling Technique (SMOTE) and two feature selection methods, combined with random forest techniques, to predict students’ final grades in their first-semester course. Keser et al. [29] provided the predictions obtained from gradient boosting, extreme gradient boosting, light gradient boosting machines, and different combinations of these algorithms as input to a super learner algorithm and used a stochastic search algorithm that optimizes the hyperparameters of the base classifier. Then, a new hybrid integrated learning algorithm (HELA) was built, which achieves the effect of good prediction performance. Qiu et al. [30] proposed the BCEP model to predict learners’ learning performance. The method considers the correlation between learning behaviors, but their feature fusion strategy is manually designed rather than automated and lacks a fine-grained feature selection mechanism.

In summary, in studies related to learning performance prediction based on learning behaviors, researchers usually analyze online learning behaviors from independent online learning behaviors or local features of behaviors. Even if online learning behaviors are classified, and features are selected, the essence is still to use independent online learning behaviors for online learning performance prediction. Few research studies have examined how to properly integrate online learning behaviors based on behavioral categories, as well as the inherent correlations and contrasts among them. Moreover, although the selection or improved utilization of learning performance prediction methods has shown good results and high efficiency for the studied problems, the methods generally have the shortcomings of low generality, complicated operation, low efficiency, and lack of intelligence. In order to achieve more accurate learning performance prediction, it is necessary to focus on the intrinsic connection between online learning behaviors and to use optimized methods for feature selection and fusion from the perspective of the EBC model to address the shortcomings of existing studies.

## 3. Method

**Figure 1:**
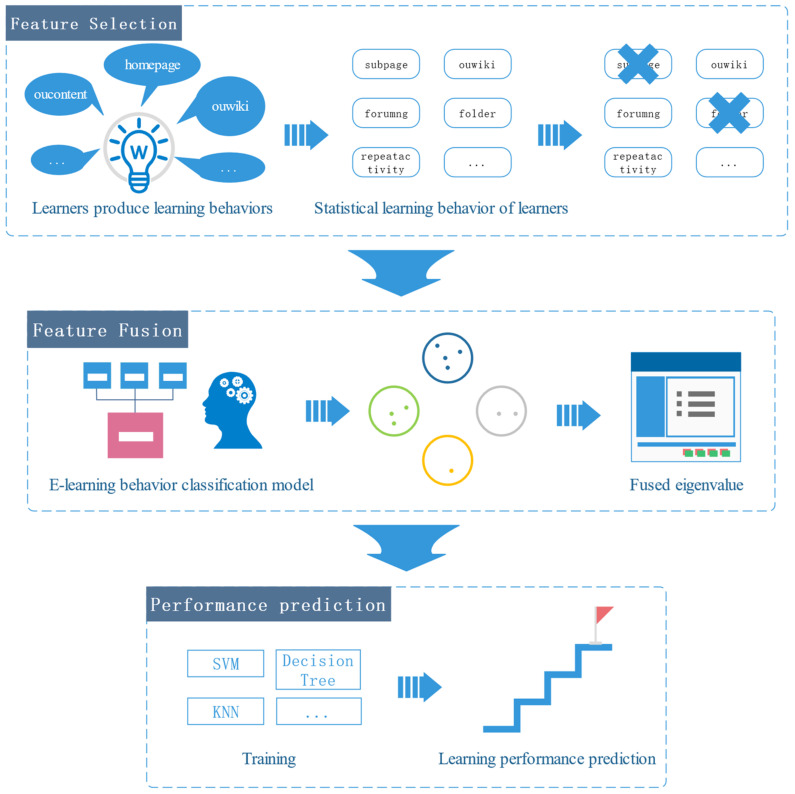
The process of self-adaptive e-learning performance prediction model.

This section describes the process of obtaining sets of learning behaviors based on an EBC model and illustrates how we can implement feature engineering. Five key components of the primary procedure of this paper’s work are shown in Figure 1: (1) Feature selection refers to screening the learning behaviors with high relevance and reducing data redundancy and the dimensionality of the features. (2) Learning behavior classification is the process of categorizing unprocessed learning behaviors using the EBC model. (3) Feature fusion refers to adaptively selecting the feature fusion method using the findings from learning behavior categorization. (4) Model training refers to anticipating learners’ e-learning performance and creating a model for predicting learning performance based on different machine learning algorithms.

### 3.1. Problem Description

By measuring, collecting, and analyzing learning behavior data to predict learning performance, it is possible to provide some instructional interventions to students once the course has begun, as well as to monitor and remind students and reduce the probability of course failure. From the standpoint of behavioral categorization, this study studies whether combinations of learning behavior sets are better predictors of academic achievement and investigates the ideal behavioral feature space. Assume there are *K* students and the e-learning behaviors generated by each student are represented by the set ${K\{S}_{1}{,S}_{2}{,\ldots ,S}_{K}\}$, where the kth student is represented by $S_{k}\left\{s_{1}{,s}_{2}{,\ldots \ldots ,s}_{n}\right\}$; $s_{n}$ stands for the nth learning behavior, and its eigenvalue is the interaction frequency of this behavior; and n=1,2,…,N is the number of e-learning behaviors (same below). Set standard e-learning behavior set $S_{k}^{\prime }{\{s}_{1}^{\prime }{,s}_{2}^{\prime }{,\ldots ,s}_{n}^{\prime }\}$ corresponding to set $S_{k}\left\{s_{1}{,s}_{2}{,\ldots \ldots ,s}_{n}\right\}$. For the feature selection stage, using the wrapper method, which takes the differential evolution algorithm as the evaluation function, conduct the initial screening of features. Next, using an e-learning classification model (EBC model), each student’s set of learning behaviors is divided into different subsets of learning behaviors. Different learning behavior subsets contain several different learning behaviors (which may also be empty sets). In this paper, we adopt the feature fusion strategy to improve the performance of the prediction model, define $D_{c_{i}}$ as the feature values of the subset $c_{i}$ after feature fusion, and construct the set of feature values $D_{c}{\{D}_{c_{1}}{,D}_{c_{2}}{,\ldots ,D}_{c_{i}}\}$ of the subset of learning behaviors.

### 3.2. E-Learning Behavior Classification Model (EBC Model)

The e-learning behavior classification model is one of the most important components of the self-adaptive e-learning performance prediction model (SA-FEM) and has a direct impact on the learning performance prediction model’s ability to anticipate outcomes. The e-learning process consists of a learning onset phase, a knowledge acquisition phase, an interactive reflection phase, and a learning consolidation phase [31]. The process that prepares learners for the formal start of e-learning is called the learning onset stage; the primary e-learning process is called the knowledge acquisition stage, which is the initial acquisition of knowledge by learners; the process of interaction and reflection between learners and teachers and peers is called the interactive reflection stage; and the learning consolidation stage refers to the procedure through which students integrate and internalize knowledge. The model focuses on the online learner and divides learning behavior into learning preparation behavior (LPB), knowledge acquisition behavior (KAB), interactive learning behavior (ILB), learning consolidation behavior (LCB), and learning consolidation behavior (LCB) based on the e-learning process:

1. Learning preparation behavior (LPB): The most fundamental learning behavior, which takes place throughout the learning preparation stage. This involves signing in to the learning platform, visiting the course page, examining the interface for the course activities, etc.;
2. Knowledge acquisition behavior (KAB): The behavior of online learners in acquiring knowledge directly, which occurs in the knowledge acquisition stage. It mainly includes participating in course activities, browsing course content resources, watching course videos, and visiting resource links;
3. Interactive learning behavior (ILB): It occurs during the interactive reflection stage and is one of the key learning behaviors in e-learning, which has been proven to have a positive effect on the continuity and effectiveness of e-learning [32]. It is manifested by participating in seminars, posting on forums, replying to forums, asking the teacher questions, etc.;
4. Learning consolidation behavior (LCB): Occurs during the learning consolidation stage and refers to the behavior of learners to reinforce their knowledge, mainly through post-lecture reflections and completion of post-lecture tests.

### 3.3. Self-Adaptive E-Learning Performance Prediction Model (SA-FEM)

Researchers frequently create prediction models of e-learning success nowadays by treating each e-learning activity as a separate predictor. However, in fact, different e-learning behaviors are potentially related to each other and can be classified into different behavioral categories based on different rules. This paper develops a model for predicting learning performance from the viewpoint of learning behavioral categories and suggests a framework based on the differential evolution algorithm [33] for predicting learning performance in online courses. The differential evolution (DE) algorithm is a simple and efficient method with the advantage of high convergence speed and good robustness, and it usually contains two phases: initialization and evolution.

The SA-FEM, as depicted in Figure 1, illustrates the whole procedure for building a learning performance prediction model using e-learning behavioral categories. The three main elements of the prediction framework are: (1) Feature selection of learner-generated learning behaviors, using a differential evolution algorithm as the evaluation function for each learning behavior to remove behaviors that contribute little to the prediction of student pass rates and obtain the core learning behaviors. (2) Fusion of features: Using the e-learning behavior categorization paradigm, the key learning behaviors are categorized, multiple subsets of learning behaviors are constructed, and then features are fused. Unlike simply fusing all the features in each subset into one feature, this paper adopts a more fine-grained fusion approach, using the differential evolution method to adaptively select which features in each subset of learning behaviors are fused and which features are discarded. The differential evolution algorithm is also applied to select which subsets of learning behaviors are enabled. The aim of this is to further use less learning behavior data to obtain better predictions of student pass rates. (3) Learner performance prediction: Seven machine learning algorithms were constructed to predict learner performance.

In the process of feature selection and feature fusion, the differential evolution algorithm is used to screen the optimal learning behavior and the optimal learning behavior fusion way. The reason why the DE algorithm is selected for feature selection and fusion is that the core of the algorithm we proposed is to explore a more fine-grained feature set, which is time-consuming work. The DE algorithm is used to search the optimal solution space of feature fusion. Compared with other genetic algorithms, the DE algorithm is not only simple, but also the convergence speed is fast and the robustness is strong. The detailed algorithm flow is shown in Algorithm 1.

In the fine-grained differential evolution model (FGDEM), the algorithm starts by setting the population size P, the dimension D of each solution, and the number of iterations of the population T. Then, the population is initialized. Each individual’s component is restricted to be generated randomly in the upper and lower bounds (rows 1 to 8); next, each individual is mutated in each iteration t: the original solution is added to a difference vector with weights. The mutated population is then randomly crossed with the original population (rows 10 to 15). The fitness of each newly generated individual is calculated. The current individual is replaced if its fitness value is higher than the original one, and the current individual is set as the optimal one if its fitness value is better than that (rows 16 to 21). Finally, the algorithm is terminated after several iterations or when it reaches the condition.
**Algorithm 1:** FGDEM: Fine-grained differential evolution Model **Input:** Population P; Dimension D; Generation T **Output:** the best vector solutions of feature fusion Δ 1.  // Initial Population. 2.  **for** *i = 1* to *P* **do** 3.   **for** *j = 1* to *D* **do** 4.    // Decide which learning behaviours to be selected. 5.    $x_{i,t}^{j}{=L}_{min}^{j}+rand\left(0,1\right)\times \left(U_{max}^{j}{-L}_{min}^{j}\right)$ 6.    Fitness Calculation. 7.   **end** 8.  **end** 9.  *t = 1*10.  *while t < T* **do**11.   **for** *i = 1* to *P* **do**12.    **for** *j = 1* to *D* **do**13.     $v_{i,t}^{j}=Mutation\left(x_{i,t}^{j}\right)$14.     $u_{i,t}^{j}=Crossover\left(x_{i,t}^{j}{,v}_{i,t}^{j}\right)$15.    **end**16.    **if** $f\left(u_{i,t}\right)<f\left(x_{i,t}\right)$ **then**17.     $x_{i,t}{=u}_{i,t}$18.     **if** $f\left(x_{i,t}\right)<f\left(\Delta \right)$ **then**19.      ${\Delta =x}_{i,t}$20.     **end**21.    **end**22.   **end**23.   *t = t + 1*24.  **end**25.  return the best solutions Δ

Similarly, the detailed algorithm flow of SA-FEM is shown in Algorithm 2, we set up two populations, initialized in the same way as the FGDEM (rows 1 to 10). We tried different mutation ways and finally compared them and found that the DE/best/1 mutation strategy is the most suitable for this prediction task. Then, the two populations are crossed separately (rows 14 to 18); since there are two populations, an optimal solution is set for each population separately, and the rest of the algorithm is consistent with the FGDEM (rows 19 to 24).
**Algorithm 2:** Self Adptive Fine-grained Feature Enginnering Model **Input:** Population P; Dimension D; Generation T **Output:** the best vector solutions of feature fusion $\Delta_{1},\Delta_{2}$ 1.  // Initial Population. 2.  **for** *i = 1* to *P* **do** 3.   **for** *j = 1* to *D* **do** 4.    // Decide which learning behaviours to be fused. 5.    ${xa}_{i,t}^{j}{=xa}_{min}^{j}+\mathrm{random}\;\mathrm{int}\;\mathrm{in}\;\left[{xa}_{min}^{j}{,xa}_{max}^{j}\right]$ 6.    // Decide which learning behaviour categories to be chosed. 7.    ${xb}_{i,t}^{j}{=xb}_{min}^{j}+rand\left(0,1\right)\times \left({xb}_{max}^{j}{-xb}_{min}^{j}\right)$ 8.    Fitness Calculation. 9.   **end**10.  **end**11.  *t = 1*12.  *while t < T* **do**13.   **for** *i = 1* to *P* **do**14.    **for** *j = 1* to *D* **do**15.     // Use DE/best/1 Mutation16.     ${va}_{i,t}^{j}{,vb}_{\left(i,t\right)}^{j}=Mutation\left({xa}_{i,t}^{j}{,xb}_{i,t}^{j}\right)$17.     ${ua}_{i,t}^{j}=Crossover\left({xa}_{i,t}^{j}{,va}_{i,t}^{j}\right)$18.    **end**19.    **if** $f\left({ua}_{i,t}{,ub}_{i,t}\right)<f\left({xa}_{i,t}{,xb}_{i,t}\right)$ **then**20.     ${xa}_{i,t}{,xb}_{i,t}{=ua}_{i,t}{,ub}_{i,t}$21.     **if** $f\left({xa}_{i,t}{,xb}_{i,t}\right)<f\left(\Delta_{1}{,\Delta }_{2}\right)$ **then**22.      $\Delta_{1}{,\Delta }_{2}{=xa}_{i,t}{,xb}_{i,t}$.23.     **end**24.    **end**25.   **end**26.   *t = t + 1*27.  **end**28. return the best solutions $\Delta_{1},\;\Delta_{2}$

#### 3.3.1. Data Pre-Processing

The prediction impact is closely proportional to the caliber of the e-learning behavior data. The data cleaning procedure on the collected learning behavior data is therefore the first stage. Since there is no standardized procedure for cleaning data, the approach to use must be chosen based on the data’s own property. The missing values, duplicate values, and anomalous values are processed. However, the e-learning behaviors that e-learning platforms capture are frequently not one-dimensional. Additionally, the values of distinct e-learning behavior data dimensions are frequently not similar; thus, this article decides to utilize Z-score normalization to converge the scale.

For the kth student, there is the set of original e-learning behaviors $X_{k}^{\prime }{\{x}_{1}^{\prime }{,x}_{2}^{\prime }{,\ldots ,x}_{n}^{\prime }\}$ and the set of standardized e-learning behaviors $X_{k}{\{x}_{1}{,x}_{2}{,\ldots ,x}_{n}\}$. $x^{\prime }{}_{n}$ denotes the nth e-learning behavior recorded by the e-learning platform, and $x_{n}$ denotes the nth e-learning behavior after standardization. Equation (1) shows the calculation of $x_{n}$.

(1) $$x_{n}=\frac{x_{n}^{\prime }{-\mu }_{n}}{\sigma_{n}}\;$$

where $\mu_{n}$ represents the mean of the nth e-learning behavior data, and $\sigma_{n}$ represents the nth e-learning behavior data variance.

#### 3.3.2. Feature Selection

In order to decrease the dimensionality of the features and increase the generalizability, operational effectiveness, and interpretability of the model, feature selection chooses the pertinent features that contribute relatively highly to the model’s training. This paper uses a differential evolution algorithm to evaluate the importance of learners’ learning behaviors, which can obtain a better feature set than filtered feature selection such as variance filtering.

First, the differential evolution algorithm is applied to feature selection, initialized to generate a population of number *M*. Each individual $X_{i}$ consists of a D-dimensional vector (*D* represents the number of learned behaviors produced by each learner), denoted as Equation (2).

(2) $$X_{i}=\left(x_{i1}{,x}_{i2}{,x}_{i3}{,\ldots ,x}_{in},\right),\;i\;\in \left[1,\;M\right]\;$$

Each component $x_{ij},\left(j\in \left[1,D\right]\right)$ is a floating-point value between 0 and 1, which is the probability value of selecting this feature. When this value is greater than the threshold value, the feature corresponding to the current component is selected, and the set threshold value is 0.5 in this paper. Each component $x_{ij}$ takes the values as shown in Equation (3), where $U_{max}$ and $L_{min}$ are the upper and lower bounds of the solution.

(3) $$x_{ij}{=L}_{min}+rand(0,1)\times \left(U_{max}{-L}_{min}\right)\;$$

The solution fitness value is calculated to select the features that contribute most to the model performance. So, the weighted sum of the F1 score, accuracy, and other metrics used to evaluate the machine learning model is used as the fitness value of the current solution and also adds the number of learning behaviors used in the fitness value calculation in order to minimize the data dimensionality and maintain better performance. The goal is to maximize the fitness value, and the fitness calculation formula is shown as Equation (4).

(4) $$f=0.3\times Accuracy+0.3\times F1\;score+0.2\times Recall+0.1\times Precsion+0.1\times Kappa+{0.1/n}_{features}$$

Then, crossover and mutation are performed, and the strategy for the gth mutation is shown as Equation (5).

(5) $$V_{i}\left(g\right){=X}_{P_{1}}\left(g\right)+F\times \left(X_{P_{2}}\left(g\right){-X}_{P_{3}}\left(g\right)\right)\;$$

where $X_{P_{1}}{,X}_{P_{2}},X_{P_{3}}$ are three random solutions in the population, and $X_{P2}\left(g\right){-X}_{P3}\left(g\right)$ is the difference vector. *F* is the scaling factor, which is usually chosen between [0, 2], and 0.5 is usually chosen.

The strategy for the gth crossover.

(6) $$U_{\left(i,j\right)}=\left\{\begin{array}{c} {Cross(x}_{ij}\left(g\right){,v}_{ij}(g)),\;rand\left(0,1\right)<cr\\ x_{ij}\left(g\right),\;else \end{array}\right.\;$$

New individuals $U_{\left(i,j\right)}$ are generated in $V_{ij}\left(g\right)$ and $X_{ij}\left(g\right)$. *cr* is the crossover rate threshold, and we choose 0.4 here. Suppose the number of randomly generated individuals is smaller than the crossover rate threshold. In that case, cross the jth component of the ith individual of the gth crossover with the component of the corresponding variant individual. In order to prevent the “early maturation” of the early population and maintain the stability of the later population, we use the adaptive crossover rate with Equation (7).

(7) $${\lambda =e}^{\left(1-T/\left(T+1-g\right)\right)}\;$$

where *T* denotes the total number of iterations, and *g* denotes the number of current iterations.

Finally, subject the new population to fitness calculation, and the selection formula for individuals in the population is shown as Equation (8).

(8) $$X_{\left(i\right)}(g+1)=\left\{\begin{array}{c} v_{i}\left(g\right),\;f\left(V_{i}\left(g\right)\right)>f\left(X_{i}\left(g\right)\right)\\ X_{i}\left(g\right),\;\;\;\;\;\;\;else \end{array}\right.\;$$

where $f\left(x\right)$ is the fitness function; the algorithm is terminated after several iterations or when it reaches the condition. The optimal solution set for feature selection is derived. Next, the current optimal set of feature solutions is used as the input for feature fusion.

#### 3.3.3. Feature Fusion

First, classify the core learning behaviors into different sets of e-learning behaviors according to the EBC model. The classification model EBC consists of learning behaviors generated by students, i.e., $EBC\left\{C_{1}{,C}_{2}{,\ldots \ldots ,C}_{n}\right\}$. After dividing the subset of e-learning behaviors, a set of n e-learning behaviors is generated. Any class of e-learning behavior subset contains a varying number of e-learning behaviors, such as $C_{1}\left\{s_{1}{,s}_{2}{,\ldots \ldots ,s}_{m}\right\}$, where $s_{m}$ denotes the mth e-learning behavior that meets the $C_{1}$ criterion.

Then, feature fusion is performed for each subset of e-learning behaviors to obtain the corresponding feature values. Taking $C_{1}\left\{s_{1}{,s}_{2}{,\ldots \ldots ,s}_{m}\right\}$ as an example, we propose a new adaptive fusion for each learning behavior subset by combining a differential evolution algorithm to decide which learning behaviors to use in that set as fused features. On the training set, the fusion strategy is determined according to the scores of each learner. We select the maximum value of each learning behavior set for passing learners and calculate the mean value for each subset of learned behaviors for failing learners. Define $D_{c_{i}}$ as the feature values after the feature fusion of the learning behavior subset $c_{i}$, which can be used to construct the learning behavior subset’s feature value set $D_{c}{\{D}_{c_{1}}{,D}_{c_{2}}{,\ldots ,D}_{c_{i}}$}. Based on the distribution of learners in the feature space, the fusion strategy is employed for the test set. Equation (9) shows the fusion strategy for the training and test sets.

(9) $$D_{c_{i}}=\lambda \;max\left\{s_{1},s_{2},\ldots ,s_{m}\right\}+\left(1-\lambda \right)\cdot \frac{\sum_{i=1}^{m}s_{i}}{m},\lambda =\left(0,1\right)$$

where $s_{i}$ is the feature value of the ith learning behavior, and $D_{C_{i}}$ denotes the feature value of a subset of e-learning behaviors of class $C_{i}$. The value of λ determines the fusion method of this student behavior class. λ=1 represents the fusion method taking the maximum strategy; λ=0 represents the fusion method taking the average strategy. For the test set, the selection of λ is derived by clustering similarity comparison, which is determined according to the neighboring training set samples of the samples.

**Figure 2:**
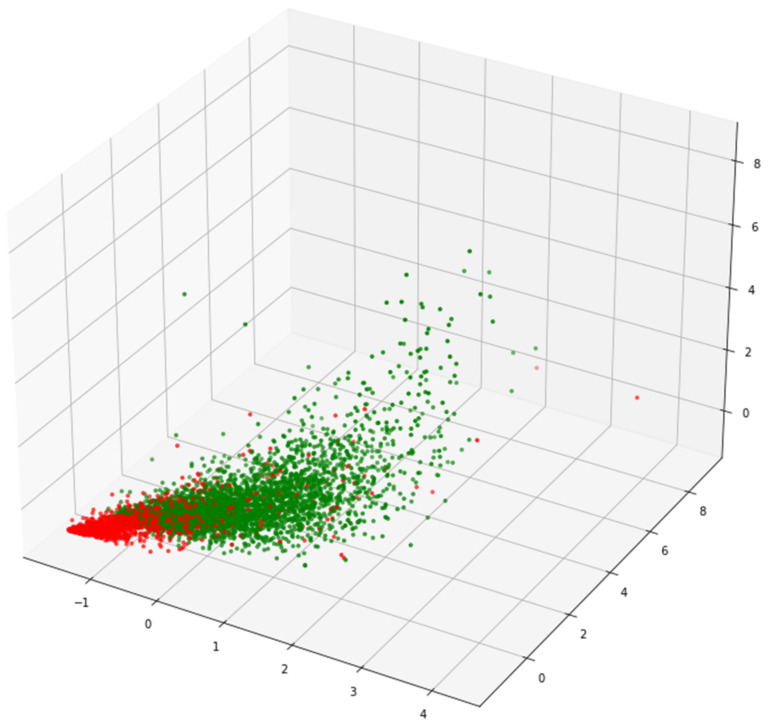
Visualizing clustering results.

Finally, we visualize and analyze learning behavior data using the principal component analysis (PCA) method. As can be seen from Figure 2, it is found that students in the red category have little difference among features. To achieve a better fusion effect, the average value of features in each category should be adopted, which means λ=1. The variability of student features in the green category is large, and if a better integration effect should be, the maximum value of features in each category should be taken, which means λ=0.

#### 3.3.4. Model Training

Traditional machine learning techniques including SVC, Naive Bayes, KNN, and Softmax are chosen for the model training session. Then, the e-learning behavior set ${K\{S}_{1}{,S}_{2}{,\ldots ,S}_{K}\}$ is used as input data to train e-learning performance prediction models. The efficiency of our suggested feature engineering techniques is evaluated after numerous iterations. To forecast the learning outcomes of online learners, the best performing e-learning performance prediction model is used.

## 4. Experimental Design

This experiment proposes a two-stage data pre-processing method: an e-learning performance prediction model based on a differential evolution algorithm for feature selection and fusion. We demonstrate the importance of data pre-processing in data analysis by analyzing experimental results. The seven machine learning methods SVC (R), SVC (L), Naive Bayes, KNN (U), KNN (D), Decision Tree, and Softmax included in the model validate the effectiveness of the feature engineering method proposed in this paper. In this paper, precision, recall, accuracy, F1 score, Kappa coefficient, and other metrics are selected as quantitative metrics to evaluate the prediction performance. The weighted sum of the above metrics is used as the fitness value of the differential evolution algorithm to fully validate the SA-FEM performance prediction model.

The hardware devices used for the experiments in this paper are an Intel Core i5-10600KF processor, 16 GB RAM, and 1 TB hard disk. The software is based on the Windows 10 operating system, and the experiments are conducted in collaboration with the Jupyter lab and PyCharm integrated working environment, where the Python interpreter version is 3.8.10.

### 4.1. Data Sources

The Open University Learning Analytics Dataset (OULAD) [34] is considered one of the most comprehensive international public datasets in terms of the diversity of e-learning data. Student demographics and student interaction data with the virtual learning environment (VLE) are included. This dataset developed by the Open University can support research in learning analytics, and analysis of this dataset can provide learners with personalized services for instructional programs. Data on e-learning behavior and academic achievement are included in the dataset for seven course modules (AAA to GGG), 22 courses, and 32,593 students. The whole dataset’s collecting process consists of three steps: collection, selection, and anonymization. A data warehouse was created with SAS technology, data that contained student information from 2013–2014 were chosen, and then an anonymization process was performed on the data. The types of design were time-series design, data integration objective, and observation design. The type of measurement was learning behavior, the type of technique was digital curation, and the type of factor was time interval. This paper selects the FFF course module with richer sample data for this experiment. The training and validation of the e-learning performance prediction model utilized the e-learning data of 5110 participants in the FFF course as the data source. While studying, learners exhibited a total of 18 e-learning behaviors, and the learning behaviors were classified according to the e-learning behavior classification model. Finally, four sets of e-learning behaviors were obtained, as shown in Table 1.

### 4.2. Experimental Design for Validation of SA-FEM

1. Experimental scheme

Four experimental groups were set up in this study to evaluate and assess the merits of feature selection and model fusion for predicting e-learning performance using a differential evolution algorithm. As input data, experimental group one employs pre-processed e-behavior data that are based on the e-learning behavior classification model. The data used in experimental group one are also used in experimental group two. It uses the differential evolution algorithm to select the effective set of learning behaviors. The final results should be consistent with experiment group one to verify the feasibility of the wrapping method using the differential evolution algorithm as the evaluation function. Experimental group three uses pre-processed data and the differential evolution algorithm to select effective learning behaviors. The fourth experimental group uses the data selected by the third experimental group and then applies the e-learning behavior classification model to fuse the behaviors further. The above experimental protocols were cross-validated using a five-fold crossover.

2. Feature selection

Experimental group one manually combined the four sets of learning behaviors according to the set of learning behaviors classified by the e-behavior classification model to obtain 15 results. The adopted method we call the e-learning performance prediction model based on manual pre-processing, referred to as the MM.

The learning behavior combination codes are shown in Table 2.

Experimental group two uses an e-learning performance prediction model based on the differential evolution algorithm referred to as the DEM. The model uses a weighted sum of predictive performance metrics as the fitness value to select the combination of the best set of learning behaviors.

The third experimental group continues to follow the idea of the differential evolution algorithm to find the optimal solution space, which is not only limited to the set of learning behaviors but also explores a more optimal feature selection strategy based on each learning behavior in a more fine-grained perspective. The adopted method is a fine-grained e-learning performance prediction model based on the differential evolution algorithm, hereafter referred to as the FGDEM.

Next, we apply the EBC model and fine-grained strategies to feature fusion, striving for a better solution space.

3. Feature fusion

**Figure 3:**
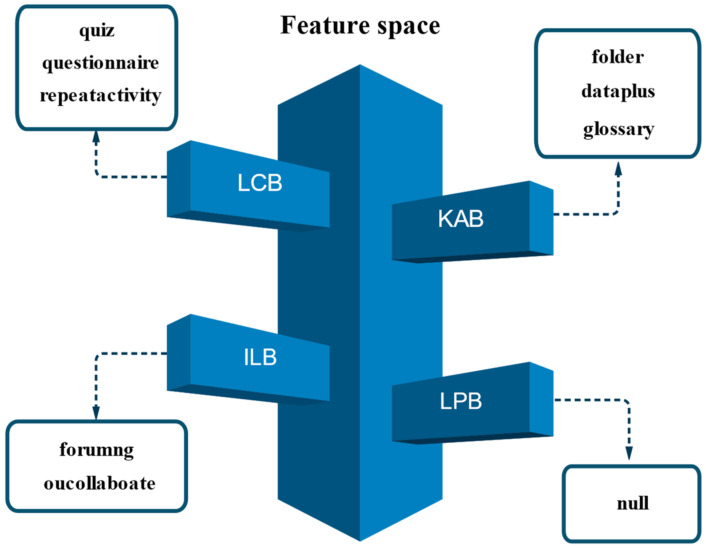
Schematic diagram of e-learning behavior classification in feature fusion.

Experimental group four constructs a self-adaptive feature-fusion-based e-learning performance prediction model called SA-FEM. Fine-grained feature fusion of behaviors is performed to obtain feature values for each subset of learning behaviors. Finally, the learner’s performance is predicted by the feature values of the subset of learning behaviors. Figure 3 shows the schematic diagram of the learning behavior classification obtained according to the EBC model with the feature selection method.

4. Mutation Strategy

We used five different mutation strategies [35] in both feature selection and feature fusion algorithms to explore which strategy has better prediction performance.

The mutation strategies are as follows:

DE/rand/1:

(10) $$v_{i}\left(g\right){=X}_{p1}\left(g\right)+F\times \left(X_{p2}\left(g\right){-X}_{p3}\left(g\right)\right)\;$$

DE/rand/2:

(11) $$v_{i}\left(g\right){=X}_{p1}\left(g\right)+F\times \left(X_{p2}\left(g\right){-X}_{p3}\left(g\right)\right)+F\times \left(X_{p4}\left(g\right){-X}_{p5}\left(g\right)\right)\;$$

DE/current to best/1:

(12) $$v_{i}\left(g\right){=X}_{i}\left(g\right)+F\times \left(X_{best}\left(g\right){-X}_{i}\left(g\right)\right)+F\times \left(X_{p1}\left(g\right){-X}_{p2}\left(g\right)\right)\;$$

DE/best/1:

(13) $$v_{i}\left(g\right){=X}_{best}\left(g\right)+F\times \left(X_{p1}\left(g\right){-X}_{p2}\left(g\right)\right)\;$$

DE/best/2:

(14) $$v_{i}\left(g\right){=X}_{best}\left(g\right)+F\times \left(X_{p1}\left(g\right){-X}_{p2}\left(g\right)\right)+F\times \left(X_{p3}\left(g\right){-X}_{p4}\left(g\right)\right)\;$$

When applying the above five strategies for model training, we record the convergence speed and overall prediction performance of each strategy and combine these two indicators to select the optimal mutation strategy.

## 5. Experimental Results and Analysis

This section gives the results of the above experiments, including the accuracy, F1 score, Kappa, recall, precision, the overall time required for feature engineering, and the optimal feature space filtered from the adaptive feature-fusion-based e-learning performance prediction model for each experimental group under the seven machine learning methods. The above data were analyzed to verify the effectiveness and feasibility of the SA-FEM and compared with existing benchmark methods.

### 5.1. Validation of SA-FEM

In this paper, four control groups (with different feature data) are designed, and seven common machine learning algorithms are used to discuss the effectiveness of the SA-FEM based on the prediction results of the prediction models.

**Figure 4:**
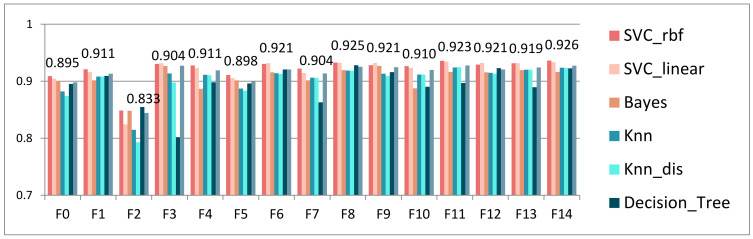
The F1 score of combination of e-learning behavior sets.

First, for the MM, the prediction metric F1 score values for each combination of data input to the algorithm are shown in Figure 4. The optimal solution is F14 which contains learning behaviors of LPB, KAB, ILB, and LCB, and the mean accuracy of the seven algorithms is 89.1%.

F2 is an interactive learning behavior category containing learning behaviors such as FU, CA, and EM. Interaction is a key behavior among many learning behaviors that affect learning outcomes in e-learning environments. The form and process of online interaction can affect motivation to some extent. E-learning interactions can also affect learners’ e-learning satisfaction and, consequently, the extent of sustained use. It can be said that e-learning interaction is the quality assurance of e-learning. Therefore, it is reasonable to use F2 interactive learning behavior for e-learning performance prediction with certain accuracy. What is recorded in the interactive learning behavior of F2 is the surface data related to FU, CA, and EM, i.e., the most basic number of interactions recorded. The quality of interaction in the FU, CA, and EM processes was not focused on and reflected in the data records. However, the mode of e-learning interaction, the depth of interaction, and the effectiveness of interaction can affect the quality of interaction, which further affects e-learning performance. The quality of the interaction cannot be determined by the superficial quantitative records alone. Such as whether the students who participated in posting or replying spoke on the relevant topics, whether the students who conducted CA shared their learning knowledge and experiences, and whether the students only shared their knowledge during the EM or went further into the knowledge-building process. The interactive learning behavior through F2 can only reflect the interactive participation, whether it is active in completing tasks or participating in group activities, and to some extent, the dynamic nature of learning. Overall, it has limitations in predicting students’ e-learning performance.

We applied the DEM in experimental group two to assess the feasibility of the wrapping method with the differential evolution algorithm as the evaluation function. The DEM automatically searches for the optimal solution in the set of learned behavior feature space, and its computational complexity is 0(n^2^). The prediction metric of the optimal solution and its corresponding feature space is shown in Table 3. The optimal solution results are consistent with the MM, and the overall convergence time of the DEM is around 9 min. While the MM exhausts the entire solution space based on manual combinatorial learning behavior, which is more labor-intensive and costs time, the DEM is superior to the MM in terms of time consumption.

The experimental results of the third experimental group are shown in Table 4, and the computational complexity of the FGDEM is 0(n^2^). Compared with the second experimental group, the prediction accuracy improved by 1%, 0.8%, 1.2%, 0.6%, 0.6%, 2.4%, and 0.7%, respectively. The improved performance of each machine learning algorithm fully demonstrates the effectiveness of the fine-grained strategy. It reduces the solution space to be searched for the subsequent feature fusion, which is meaningful.

The experimental results of the fourth experimental group are shown in Table 5. The computational complexity of the SA-FEM is also 0(n^2^). Moreover, all of its metrics have a greater improvement than those of the FGDEM. Among the seven machine learning algorithms, accuracy improved by 3.8%, 4.5%, 5.2%, 6.1%, 6%, 8.2%, and 6.5%, respectively. Although the solution space of this experimental group is not as large as that of the FGDEM, it takes a little time to execute the feature fusion strategy, so the overall convergence time of the algorithm is a little longer than that of the FGDEM. However, the FGDEM effectively reduces the feature space to be searched, implying a contribution to the reduction in time spent in the SA-FEM. The optimal solution uses only three features (after feature fusion) to achieve a 6.42% improvement in accuracy compared to the MM average.

The FFF course chosen for the experiment is a course in the STEM field. It records mostly data generated from learning activities conducted in the course, which can also be classified into the four learning behavior categories mentioned above according to the learning behavior classification framework. However, it is not reasonable and accurate to use HP, PG, and SP, the most basic online platform login operations in the learning readiness behavior category, for learning performance prediction. HP, PG, and SP are the only necessary steps to access subsequent operations, such as browsing content and accessing resources. In a certain sense, they are homogeneous and do not reflect the state of student learning or achievement. Thus the data were subjected to an experimental adaptive feature fusion operation to derive an optimal solution feature space that excels in various performance aspects, such as accuracy, Kappa, F1 score, and other metrics. This feature space was selected for learning performance prediction only for three behavioral categories that mainly reflect the e-learning process: knowledge acquisition behavior, interactive learning behavior, and learning consolidation behavior.

Compared with the MM, DEM, and FGDEM, this feature space can achieve higher prediction accuracy by only three learned behavioral features (after feature fusion). The three behavioral features, DA, FD, and GS, all belong to the category of knowledge acquisition behaviors. They are specifically interpreted as supplemental data, opening folders, and accessing glossaries of terms. DA and GS are the basic operations students perform when learning on the platform. FD is a prerequisite for subsequent resource access and acquisition. The combination of these three can reflect some extent, whether students browse the platform’s resources, and their propensity to perform knowledge learning and has the advantage of a large amount of accessible data.

FU and CA were chosen over EM in the interactive learning behavior category because although EM enhances the facial emotional communication experience through technology, communication in video form may expose students to a more uncontrollable environment. According to the spiral of silence theory, people are often influenced by a “fear of isolation” when expressing their ideas and opinions. When they find that no one or very few people pay attention to a certain point of view (and sometimes they are attacked by a group), they remain silent even if they agree with it. Students’ real thoughts are more difficult to capture during this interactive exchange, and prediction of learning performance in this way can be ineffective. In contrast, the number of posts and replies and textual information in forum discussions have some value, so predictions can be made by obtaining data and mining and analyzing textual values [36]. Collaborative communication is an important channel for internalizing learning content into self-cognitive structures and developing higher-order thinking. Through teacher–student and student–student communication and interaction, it is beneficial for both parties to interact with information and maintain emotional needs. The content and level of collaborative communication indicate students’ understanding and engagement in learning. These two data reflect the mastery of students’ knowledge and their participation, making it more accurate to predict learning performance with this interactive communication status data.

Learning consolidation behavior is a step students take to consolidate and reinforce their knowledge after learning something new. QN refers to participation in questionnaire feedback, which helps us understand students’ emotional states and further analyze the strength of their motivation for deeper learning. QZ refers to the stage tests that learners take. The test results not only help us see the effectiveness of students’ stage learning but also provide a basis for students to reflect on their stage learning results. RP refers to repetitive activities. The data of repeat activity can, to a certain extent, reflect the continuity and frequency of learning, i.e., the continuity of students’ review of knowledge. These three data quantify the student learning consolidation process in different directions and help make a more comprehensive prediction of learning performance.

The above three types of learning behaviors describe the three stages of learning new knowledge, interacting and collaborating, and reviewing and consolidating that students go through when they engage in e-learning. The relevant behavioral features from different perspectives are used to deeply and comprehensively shape the learning portrait of students, which is conducive to more accurate learning performance prediction.

**Figure 5:**
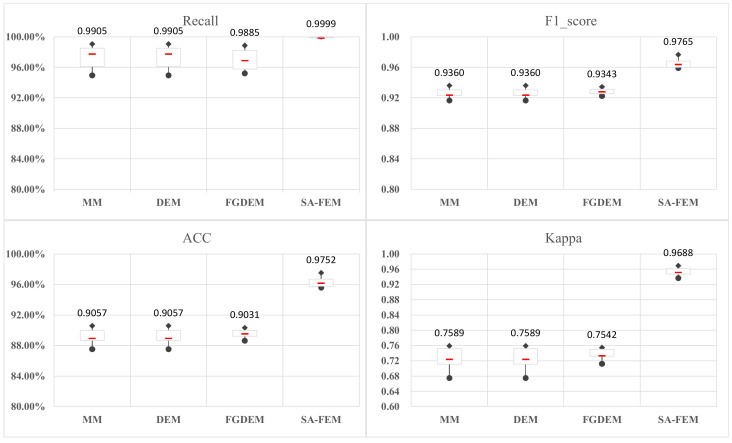
Ablation experiments.

As shown in Figure 5, a longitudinal comparison of the prediction performance results of the four models for the FFF dataset shows that the results of the MM and DEM are consistent. The FGDEM implements a fine-grained feature selection strategy to make all four prediction performance metrics of the seven machine learning algorithms have a better level. The box plot can intuitively observe that the scattering range of the FGDEM is lower than the MM and DEM, which shows that the FGDEM algorithm performs better from the side. The prediction performance metrics of the SA-FEM algorithm are even higher than the MM, DEM, and FGDEM algorithms. It can be seen from the figure that the SA-FEM has a smaller scattering range and lower bias compared with the MM, DEM, and FGDEM. The above comparison fully demonstrates the superiority of our proposed SA-FEM.

### 5.2. Overall Evaluation of Wrapper Method with Differential Evolution Algorithm as an Evaluation Function

The results of ten runs of the FGDEM are shown in Table 6. It can be seen that the degree of fluctuation of each metric is very small. The variance of precision, recall, accuracy, F1 score, and Kappa in the results of ten runs is $1.818\times 10^{-6},2.491\times 10^{-6},5.03\times 10^{-7},2.06\times 10^{-7},4.15\times 10^{-6}$, respectively. The results of multiple experimental runs show that our proposed algorithm has good stability.

**Figure 6:**
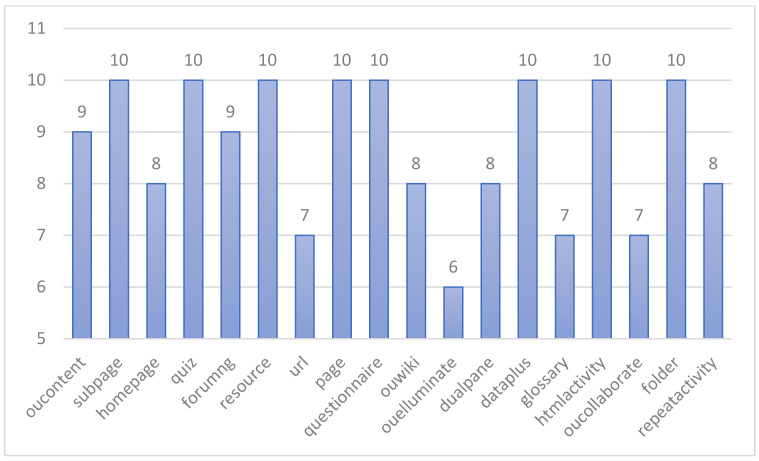
Diagram of feature space of ten FGDEM runs.

The distribution of the solution space derived from ten runs of the SA-FEM with the distribution based on the set of learning behaviors is shown in Figure 6. It can be visually observed that HA, CT, WK, RS, UR, and EM are less frequently selected as learning performance predictors in the feature space. When establishing the optimal solution space, it is even more necessary to select suitable and reasonable behavioral features from multiple perspectives. HA, CT, WK, RS, and UR are knowledge acquisition behaviors that are largely repetitive in a behavioral sense and are more influenced by other factors, so they have a lower chance of being selected or selected simultaneously. Compared to FU and CA, the effect of EM on students is more unpredictable, so it is less likely to be used as a factor in constituting the feature space. In addition, it can be seen from the histogram that the distribution of the solution space from the ten runs of the SA-FEM has a commonality with the optimal solution space in that none of them contains the learning readiness behavior category. In the optimal solution, DA, FD, GS, FU, CA, QN, QZ, and RP belong to behavioral features that occur with high frequency in the results of the ten-run distribution. The two can corroborate each other and improve persuasiveness.

The metric results of ten SA-FEM runs are shown in Table 7, and the average accuracy of the optimal solution is 96.11%. Compared with the FGDEM, adding the feature fusion strategy can improve the model’s prediction performance to a larger extent. Moreover, the variance of each indicator after ten runs shows that the dispersion is very small, which fully proves the high stability of the proposed algorithm.

**Figure 7:**
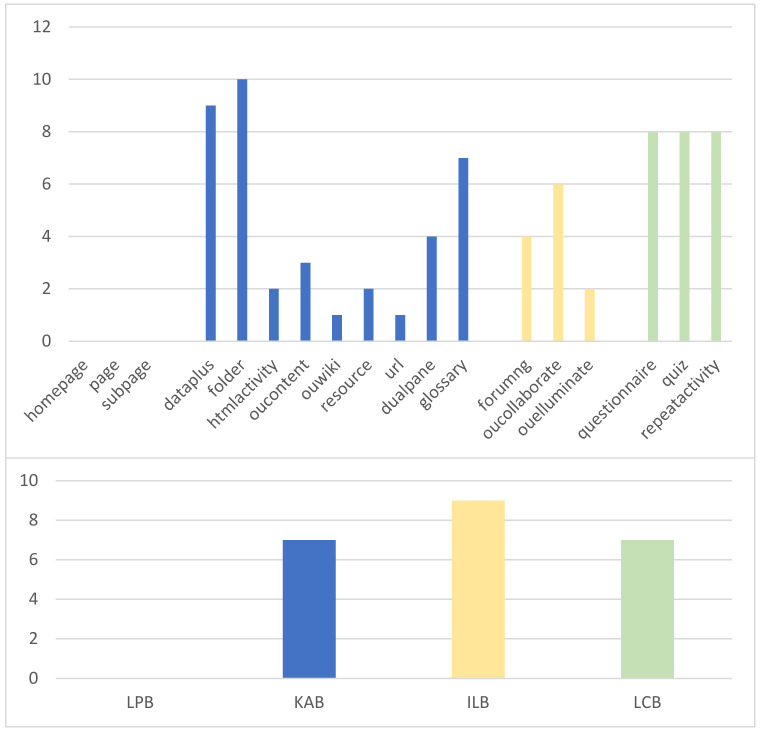
Diagram of accumulative feature space of SA-FEM 10-time running results.

The distribution of the solution space derived from ten runs of the SA-FEM with the distribution based on the set of learning behaviors is shown in Figure 7. It can be visually observed that HA, CT, WK, RS, UR, and EM are less frequently selected as learning performance predictors in the feature space. When establishing the optimal solution space, it is even more necessary to select suitable and reasonable behavioral features from multiple perspectives. HA, CT, WK, RS, and UR are knowledge acquisition behaviors that are largely repetitive in a behavioral sense and are more influenced by other factors, so they have a lower chance of being selected or selected simultaneously. Compared to FU and CA, the effect of EM on students is more unpredictable, so it is less likely to be used as a factor in constituting the feature space. In addition, it can be seen from the histogram that the distribution of the solution space from the ten runs of the SA-FEM has a commonality with the optimal solution space in that none of them contains the learning readiness behavior category. In the optimal solution, DA, FD, GS, FU, CA, QN, QZ, and RP belong to behavioral features that occur with high frequency in the results of the ten-run distribution. The two can corroborate each other and improve persuasiveness.

The comparison between the predictive performance metrics of the results of ten runs and the analysis of the frequently selected learning behaviors fully demonstrate the effectiveness of our proposed feature engineering approach using a differential evolution algorithm as an evaluator and a weighted value of the predictive performance metrics as the fitness value. It also provides a new perspective for the evaluation of e-learning behaviors.

We compared e-learning performance prediction methods using FFF course data from the OULAD dataset, as shown in Table 8. Our proposed method is an improvement over existing benchmark methods, using machine learning algorithms and evolution algorithms that are less resource-intensive than the more resource-intensive deep learning methods employed by most methods. Deep learning methods are not interpretable, whereas our approach allows for an educational theoretical analysis of learner behavior. Furthermore, without loss of generality, our main work focuses on feature engineering, where the upper limit of machine learning is determined by feature engineering, not by machine learning itself. So our proposed method is more portable than other methods. Although the improvement of accuracy of our proposed method is not much compared with Qiu’s method, we use a fine-grained feature selection method with an adaptive feature fusion method, which is more efficient compared with the manual search of the optimal solution space; and we have to reduce one more dimension in the feature dimensionality reduction.

### 5.3. Comprehensive Analysis of Different Mutation Strategies

We compare the performance of different mutation strategies and explore the convergence speed of different mutation strategies. After comprehensive comparison, it is found that the DE/best/1 strategy has a good effect on this task and the convergence speed of the algorithm is also fast.

**Figure 8:**
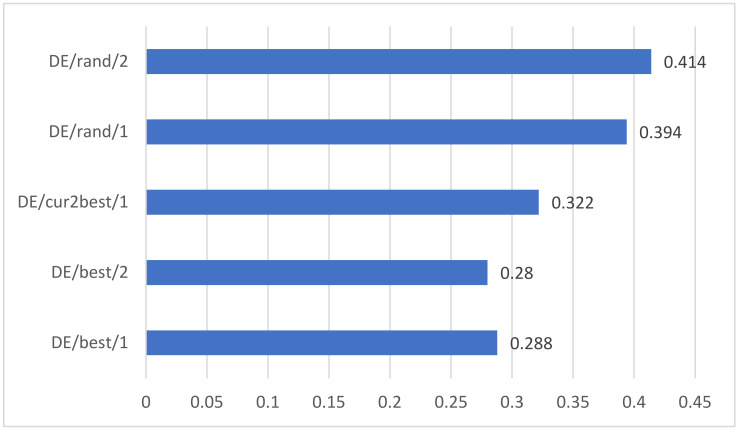
Convergence rate of five mutation strategies after unified dimension.

Figure 8 shows the iteration number required for the convergence of the five mutation strategies after the unified dimension. It can be seen that the DE/best/1 strategy has the fastest convergence speed. Next is DE/best/2, and the DE/rand/2 strategy has the slowest convergence speed.

**Figure 9:**
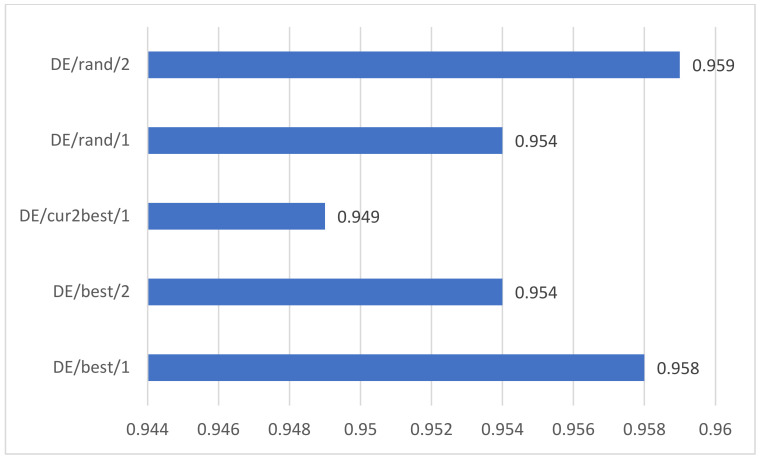
The prediction accuracy of the model using five different mutation strategies.

At the same time, we compare the performance of five mutation strategies on student performance prediction. As shown in Figure 9, we can see that the best mutation strategy is DE/rand/2, with a prediction accuracy of 95.9%. However, in combination with the convergence rate of Figure 8, we find that DE/best/1 converges much faster than DE/rand/2 when the accuracy decreases by only 0.001%, so our final choice of mutation strategy is DE/best/1.

## 6. Conclusions

One efficient method for guaranteeing the caliber of e-learning is the learning performance prediction model. A popular area of research in the world of e-learning is how to construct learning performance prediction models with high generality and high accuracy. This work, from the viewpoint of learner learning behavior categorization, offers a novel adaptive feature-fusion-based e-learning performance prediction model. To the conventional approach, it adds a learning behavior feature fusion mechanism. Experimental results on the OULAD dataset show that the model proposed in this paper significantly outperforms traditional learning performance prediction models. This framework produces a precise and reliable learning performance prediction model. A novel workable way to quantitatively assess the benefits and drawbacks of e-learning behavior classification techniques is offered by the follow-up tests, which show that the SA-FEM introduced in this study is efficient and superior to existing e-learning behavior classification methods. The model proposed in this paper is more interpretable than those based on deep learning methods. The model can still effectively predict students’ learning performance due to its adaptive nature in the future when e-learning platforms can collect more detailed learning behavior data. However, there are still some limitations. The whole process of our method is a little time consuming, and the weights of the fitness function need manual tuning.

In our upcoming study, we will create learning performance prediction models using the methodology presented in this research for different e-learning platforms and further optimize the prediction models in this paper by recording and analyzing the performance of these prediction models in the process of actual use. At the same time, we believe that the e-learning prediction aim should be more varied. Comparable methods will also be employed to forecast e-learning sentiment in addition to the e-learning performance prediction discussed in this work to improve online supervision and early warning through the prediction findings from several perspectives, as well as to ensure the effectiveness of the e-learning system and to guarantee the learning quality of online students.

## Figures and Tables

**Table 1:** E-learning behavior and coding of FFF courses.

	Behavior Sets	Behavior Codes	E-Learning Behavior	Behavior Interpretation
1	LPB	HP	homepage	Access the homepage
2		PG	page	Enter the course interface
3		SP	subpage	Enter the course subinterface
4	KAB	DA	dataplus	Supplementary materials
5		FD	folder	Open the folder
6		HA	htmlactivity	Web activity
7		CT	oucontent	Download platform resources
8		WK	ouwiki	Search on Wikipedia
9		RS	resource	Search platform resources
10		UR	url	Access the link
11		DU	dualpane	Access to double Windows
12		GS	glossary	Access to the glossary
13	ILB	FU	forumng	BBS discussion
14		CA	oucollaborate	Collaborative communication
15		EM	ouelluminate	Simulation Seminar
16	LCB	QN	questionnaire	Participate in questionnaire feedback
17		QZ	quiz	Quiz
18		RP	repeatactivity	Repeat activities

**Table 2:** Combination of online learning behavior sets.

Input Indicator Number	Behavior Set Number
F0	LPB
F1	KAB
F2	ILB
F3	LCB
F4	LPB, KAB
F5	LPB, ILB
F6	LPB, LCB
F7	KAB, ILB
F8	KAB, LCB
F9	ILB, LCB
F10	LPB, KAB, ILB
F11	LPB, KAB, LCB
F12	LPB, ILB, LCB
F13	KAB, ILB, LCB
F14	LPB, KAB, ILB, LCB

**Table 3:** DEM’s optimal solution and optimal feature space.

Method	Precision	Recall	Accuracy	F1 Score	Kappa	Feature Space	Behavior Classes
SVC_rbf	0.886	0.989	0.904	0.935	0.753	HP, PG, SP, DA, FD, HA, CT, WK, RS, UR, DU, GS, FU, CA, EM, QN, QZ, RP	LPB, KAB, ILB, LCB
SVC linear	0.895	0.974	0.903	0.933	0.755		
Bayes	0.858	0.983	0.875	0.916	0.674		
Knn	0.890	0.958	0.888	0.922	0.721		
Knn_dis	0.890	0.958	0.888	0.922	0.721		
Decision Tree	0.858	0.976	0.870	0.913	0.661		
Softmax	0.906	0.946	0.895	0.926	0.743		

**Table 4:** FGDEM’s optimal solution and optimal feature space.

Method	Precision	Recall	Accuracy	F1 Score	Kappa	Feature Space	Behavior Classes
SVC_rbf	0.902	0.986	0.914	0.942	0.774	CT, SP, HP, QZ, RS, PG, QN, EM, DU, DA, HA, FD, RP	LPB(part), KAB(part), ILB(part), LCB
SVC linear	0.909	0.973	0.911	0.940	0.771		
Bayes	0.875	0.982	0.887	0.926	0.698		
Knn	0.904	0.953	0.894	0.928	0.731		
Knn_dis	0.904	0.953	0.894	0.928	0.731		
Decision Tree	0.877	0.970	0.882	0.921	0.687		
Softmax	0.921	0.944	0.902	0.932	0.757		

**Table 5:** SA-FEM’s optimal solution and optimal feature space.

Method	Precision	Recall	Accuracy	F1 Score	Kappa	Feature Space	Behavior Classes
SVC_rbf	0.960	1.000	0.962	0.964	0.951	DA, FD, GS, FU, CA, QN, QZ, RP	KAB(part) ILB(part) LCB
SVC linear	0.956	1.000	0.958	0.960	0.945		
Bayes	0.974	1.000	0.975	0.977	0.969		
Knn	0.970	0.998	0.970	0.971	0.964		
Knn_dis	0.960	0.996	0.958	0.959	0.950		
Decision Tree	0.953	1.000	0.956	0.960	0.937		
Softmax	0.963	1.000	0.964	0.965	0.959		

**Table 6:** Metrics result of ten FGDEM runs.

	Precision	Recall	Accuracy	F1_Score	Kappa
1	0.891	0.969	0.896	0.928	0.738
2	0.888	0.975	0.896	0.929	0.737
3	0.891	0.972	0.897	0.929	0.742
4	0.888	0.973	0.896	0.929	0.737
5	0.892	0.971	0.897	0.929	0.743
6	0.891	0.970	0.896	0.929	0.740
7	0.890	0.972	0.897	0.929	0.740
8	0.889	0.971	0.895	0.928	0.736
9	0.890	0.971	0.896	0.929	0.739
10	0.890	0.971	0.896	0.929	0.739
Var	$1.818\times 10^{-6}$	$2.491\times 10^{-6}$	$5.03\times 10^{-7}$	$2.06\times 10^{-7}$	$4.15\times 10^{-6}$

**Table 7:** Metrics result of ten SA-FEM runs.

	Precision	Recall	Accuracy	F1_Score	Kappa
1	0.9599	0.9990	0.9603	0.9618	0.9524
2	0.9666	0.9992	0.9671	0.9685	0.9599
3	0.9714	0.9992	0.9723	0.9742	0.9626
4	0.9595	0.9983	0.9598	0.9622	0.9478
5	0.9590	0.9750	0.9423	0.9510	0.8985
6	0.9657	0.9986	0.9677	0.9717	0.9469
7	0.9568	0.9992	0.9576	0.9593	0.9491
8	0.9577	0.9781	0.9547	0.9519	0.9028
9	0.9596	0.9994	0.9609	0.9629	0.9508
10	0.9686	0.9975	0.9681	0.9704	0.9563
avg	0.9625	0.9944	0.9611	0.9634	0.9427
Var	0.000008	0.000089	0.000068	0.00003	0.000484

**Table 8:** Comparison of accuracy with existing benchmark methods.

	Accuracy
He	80.00%
Zheng	87%
Aurora	90.37% (avg)
Naif	95.23%
Qiu	95.4% (avg)
Ours	95.80%

## Data Availability

The public dataset that we use in this paper can be founded at https://analyse.kmi.open.ac.uk/open_dataset (accessed on 18 October 2022).

## References

[1] Adedoyin O.B., Soykan E. (2020). COVID-19 pandemic and online learning: The challenges and opportunities. Interactive Learn. Environ..

[2] Xiang L., Stillwell J., Burns L., Heppenstall A. (2020). Measuring and assessing regional education inequalities in China under changing policy regimes. Appl. Spat. Anal. Policy.

[3] Feng W., Tang J., Liu T.X. Understanding dropouts in MOOCs. Proceedings of the AAAI Conference on Artificial Intelligence.

[4] Marras M., Vignoud J.T.T., Kaser T. Can feature predictive power generalize? benchmarking early predictors of student success across flipped and online courses. Proceedings of the 14th International Conference on Educational Data Mining.

[5] He Y., Chen R., Li X., Hao C., Liu S., Zhang G., Jiang B. (2020). Online at-risk student identification using RNN-GRU joint neural networks. Information.

[6] Hao J., Gan J., Zhu L. (2022). MOOC performance prediction and personal performance improvement via Bayesian network. Educ. Inf. Technol..

[7] Wang C. (2021). Analysis of students’ behavior in english online education based on data mining. Mob. Inf. Syst..

[8] Mai T.T., Bezbradica M., Crane M. (2022). Learning behaviours data in programming education: Community analysis and outcome prediction with cleaned data. Future Gener. Comput. Syst..

[9] Fan Y., Wang Q. (2018). Prediction of academic performance and risk: A review of literature on predicative indicators in learning analytics. Distance Educ. China.

[10] Espinoza A.M., Taut S. (2020). Gender and psychological variables as key factors in mathematics learning: A study of seventh graders in Chile. Int. J. Educ. Res..

[11] Erickson V.L. Data-driven models to predict student performance and improve advising in computer science. Proceedings of the International Conference on Frontiers in Education: Computer Science and Computer Engineering (FECS).

[12] Zafari M., Sadeghi-Niaraki A., Choi S.-M., Esmaeily A. (2021). A Practical Model for the Evaluation of High School Student Performance Based on Machine Learning. Appl. Sci..

[13] Yu C.-H., Wu J., Liu A.-C. (2019). Predicting learning outcomes with MOOC clickstreams. Educ. Sci..

[14] Loginova E., Benoit D.F. Embedding Navigation Patterns for Student Performance Prediction. Proceedings of the 14th International Conference on Educational Data Mining.

[15] Yoo J.E., Rho M. LMS Log Data Analysis from Fully-Online Flipped Classrooms: An Exploratory Case Study via Regularization. Proceedings of the 14th International Conference on Educational Data Mining.

[16] Zheng Y., Gao Z., Wang Y., Fu Q. (2020). MOOC dropout prediction using FWTS-CNN model based on fused feature weighting and time series. IEEE Access.

[17] Wen Y., Tian Y., Wen B., Zhou Q., Cai G., Liu S. (2019). Consideration of the local correlation of learning behaviors to predict dropouts from MOOCs. Tsinghua Sci. Technol..

[18] Akram A., Fu C., Li Y., Javed M.Y., Lin R., Jiang Y., Tang Y. (2019). Predicting students’ academic procrastination in blended learning course using homework submission data. IEEE Access.

[19] Khan A., Ghosh S.K., Ghosh D., Chattopadhyay S. (2021). Random wheel: An algorithm for early classification of student performance with confidence. Eng. Appl. Artif. Intell..

[20] Abidi S.M.R., Zhang W., Haidery S.A., Rizvi S.S., Riaz R., Ding H., Kwon S.J. (2020). Educational sustainability through big data assimilation to quantify academic procrastination using ensemble classifiers. Sustainability.

[21] Hooshyar D., Pedaste M., Yang Y. (2019). Mining educational data to predict students’ performance through procrastination behavior. Entropy.

[22] Figueroa-Cañas J., Sancho-Vinuesa T. (2020). Early prediction of dropout and final exam performance in an online statistics course. IEEE Rev. Iberoam. Tecnol. Aprendiz..

[23] Esteban A., Romero C., Zafra A. (2021). Assignments as Influential Factor to Improve the Prediction of Student Performance in Online Courses. Appl. Sci..

[24] Aydoğdu Ş. (2020). Predicting student final performance using artificial neural networks in online learning environments. Educ. Inf. Technol..

[25] Mubarak A.A., Cao H., Zhang W., Zhang W. (2021). Visual analytics of video-clickstream data and prediction of learners’ performance using deep learning models in MOOCs’ courses. Comput. Appl. Eng. Educ..

[26] Song X., Li J., Sun S., Yin H., Dawson P., Doss R.R.M. (2020). SEPN: A sequential engagement based academic performance prediction model. IEEE Intell. Syst..

[27] Hasan R., Palaniappan S., Mahmood S., Abbas A., Sarker K.U., Sattar M.U. (2020). Predicting student performance in higher educational institutions using video learning analytics and data mining techniques. Appl. Sci..

[28] Bujang S.D.A., Selamat A., Ibrahim R., Krejcar O., Herrera-Viedma E., Fujita H., Ghani N.A.M. (2021). Multiclass prediction model for student grade prediction using machine learning. IEEE Access.

[29] Keser S.B., Aghalarova S. (2022). HELA: A novel hybrid ensemble learning algorithm for predicting academic performance of students. Educ. Inf. Technol..

[30] Qiu F., Zhang G., Sheng X., Jiang L., Zhu L., Xiang Q., Jiang B., Chen P.-k. (2022). Predicting students’ performance in e-learning using learning process and behaviour data. Sci. Rep..

[31] Yueya S. (2015). Characteristics of online learning behavior of Distance learners in the Open University. China Educ. Technol..

[32] Yu H., Harper S., Vigo M. (2021). Modeling micro-interactions in self-regulated learning: A data-driven methodology. Int. J. Hum.-Comput. Stud..

[33] Parouha R.P., Verma P. (2022). A systematic overview of developments in differential evolution and particle swarm optimization with their advanced suggestion. Appl. Intell..

[34] Kuzilek J., Hlosta M., Zdrahal Z. (2017). Open university learning analytics dataset. Sci. Data.

[35] Ahmad M.F., Isa N.A.M., Lim W.H., Ang K.M. (2021). Differential evolution: A recent review based on state-of-the-art works. Alex. Eng. J..

[36] Onan A. (2021). Sentiment analysis on massive open online course evaluations: A text mining and deep learning approach. Comput. Appl. Eng. Educ..

